# Supplementary material for: Heterogeneity of IL-15-expressing mesenchymal stromal cells controls natural killer cell development and immune cell homeostasis
Source: Nat Commun. 2025 Jul 1;16:5949. doi: 10.1038/s41467-025-61231-0 (PMC12218584; doi:10.1038/s41467-025-61231-0)
Supplement: Supplementary file 1 — Supplementary Information [file 41467_2025_61231_MOESM1_ESM.pdf]

Heterogeneity of IL-15-expressing mesenchymal stromal cells controls natural killer cell development and immune cell homeostasis

Carmen Stecher[1], Romana Bischl[1], Anna Schmid-Böse[1], Stefanie Ferstl[1], Elisabeth Potzmann[1], Magdalena Frank[1], Nina Braun[1], Matthias Farlik[2], Richard A. Flavell[3], Dietmar Herndler-Brandstetter[1,*]

[1] Center for Cancer Research, Medical University of Vienna and Comprehensive Cancer Center, 1090 Vienna, Austria

[2] Department of Dermatology, Medical University of Vienna and Comprehensive Cancer Center, 1090 Vienna, Austria

[3] Department of Immunobiology, Yale School of Medicine, New Haven, CT 06520, USA; Howard Hughes Medical Institute, Yale School of Medicine, New Haven, CT 06520, USA

* Correspondence: dietmar.herndler-brandstetter@meduniwien.ac.at

This file contains:

Supplementary Figures 1-10

Supplementary Tables 1-3

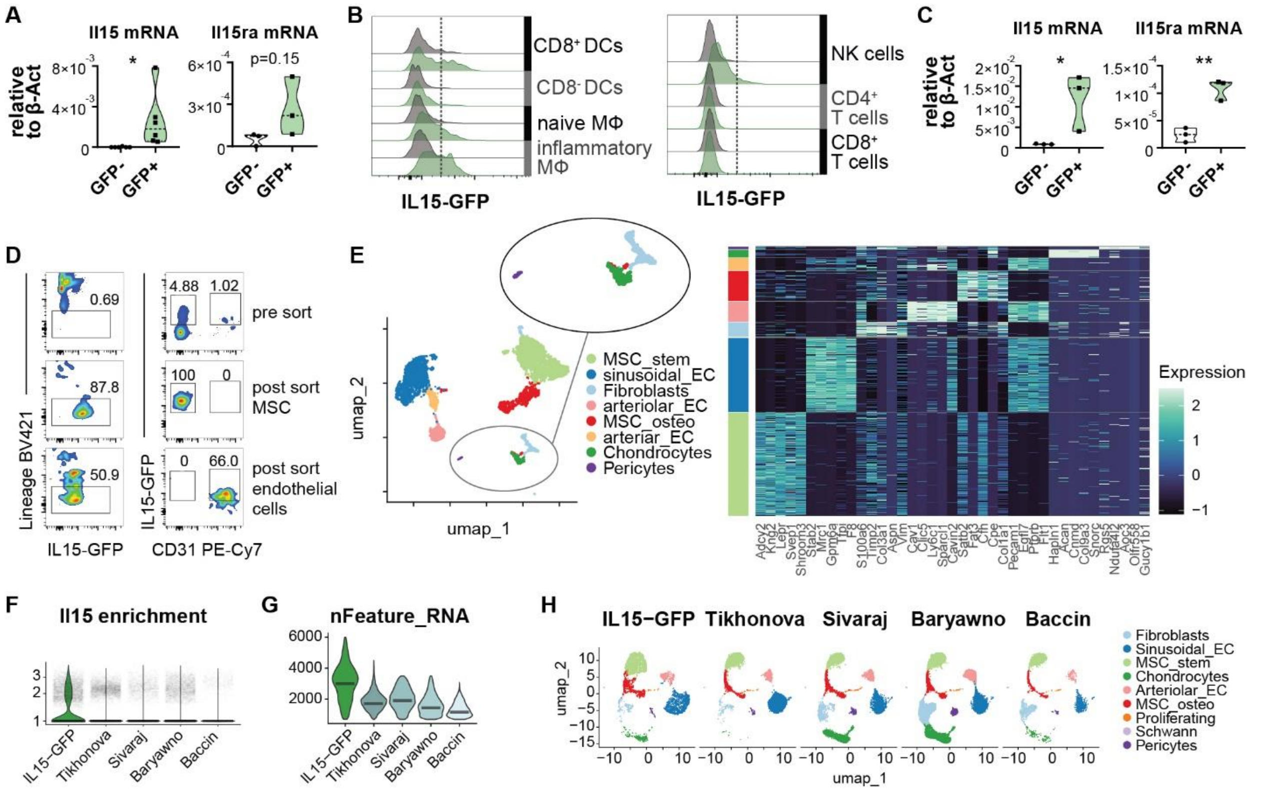

Supplementary Figure 1. Characterization of $Il15^{GFP}$ homozygous knockin reporter mice, related to Figure 1. (A) *Il15* and *Il15ra* mRNA expression in $Il15^{GFP-}$ versus $Il15^{GFP+}$ FACS-sorted stromal cells (2^-dCT versus *β-Act*) (n=3-6 biological replicates from 3-6 independent experiments). **(B)** $Il15^{GFP}$ expression in $CD8^+$ DCs ($CD45^+CD11c^+CD8^+$), $CD8^-$ DCs ($CD45^+CD11c^+CD8^-$), naïve macrophages ($CD45^+CD11b^+F4/80^+Ly6C^-$), inflammatory macrophages ($CD45^+CD11b^+F4/80^+Ly6C^+$), NK cells ($CD45^+CD3^-NK1.1^+$), $CD4^+$ and $CD8^+$ T cells ($CD45^+CD3^+NK1.1^-CD4^+/CD8^+$) in the spleen of $Il15^{GFP+/+}$ mice compared to fluorescence minus one controls (C57BL/6J WT mice). **(C)** *Il15* and *Il15ra* mRNA expression in $Il15^{GFP-}$ versus $Il15^{GFP+}$ BM macrophages (2^{-dCT} versus *β-Act*) (n=3 biological replicates from 2 independent experiments). **(D)** Representative quality control of previously sorted $Il15^{GFP+}$ stromal cells ($CD31^-$ stromal cells or $CD31^+$ ECs) in purity mode. **(E)** UMAP projection of the $Il15^{GFP+}$ scRNA-seq dataset shown in Figure 1F with unsupervised clustering (left) and heatmap of the top5 expressed genes per cluster (right). **(F)** ViolinPlot showing *Il15* expression in own versus public scRNA-seq dataset. **(G)** Violin Plot showing the average number of features (nFeature_RNA) between the $Il15^{GFP+}$ scRNA-seq dataset and the indicated public datasets. **(H)** UMAP projection of the Harmony-integrated, gene label harmonized meta-dataset (including $Il15^{GFP+}$ and the four indicated public datasets) used in F. Dot plots show mean ± SEM with each dot representing a biological replicate. Numbers above the dot plots indicate *P* values from unpaired two-tailed Student's t-tests. * $P < 0.05$, ** $P < 0.01$.

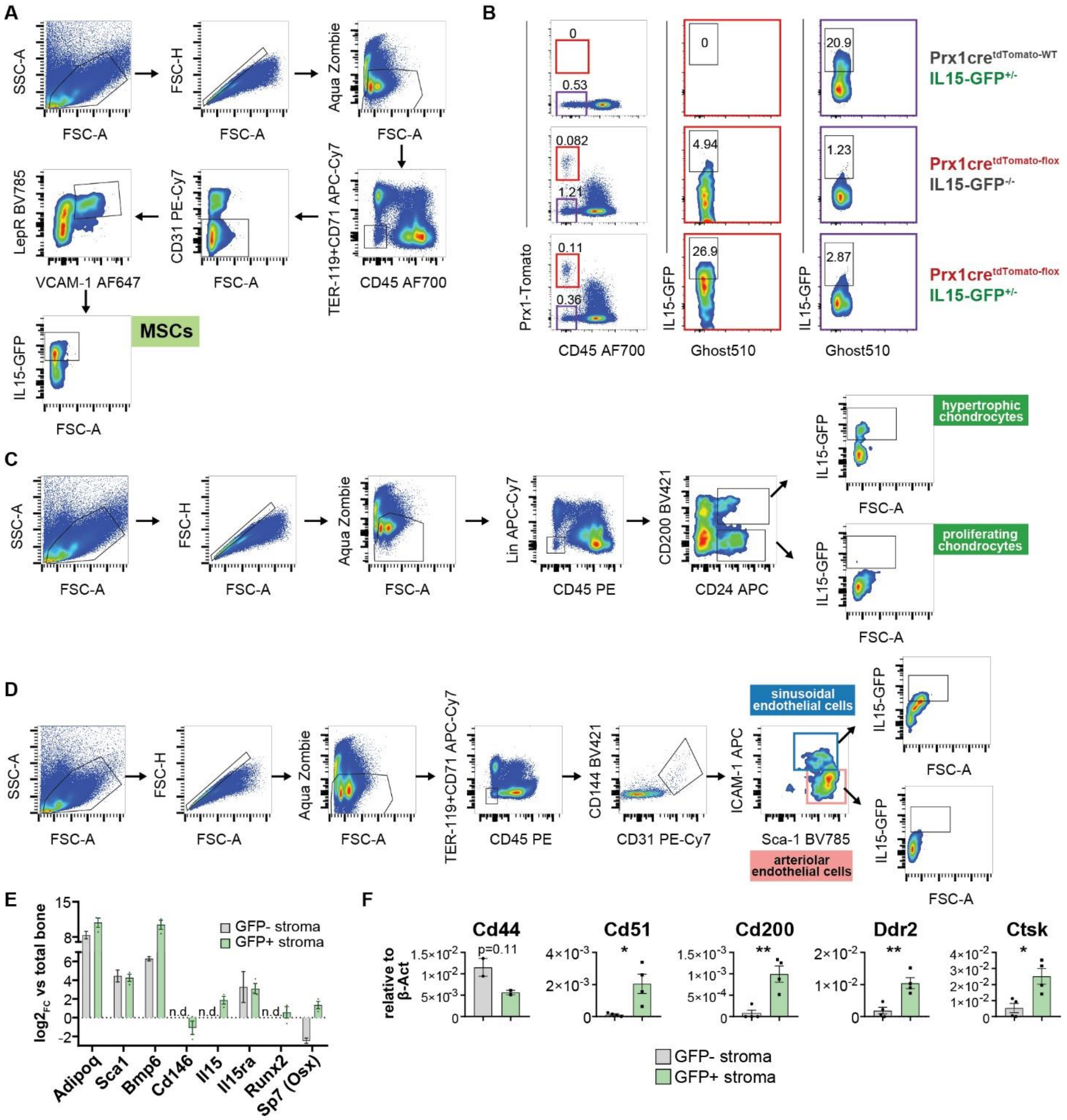

Supplementary Figure 2. Characterization of Il15GFP BM stromal cells, related to Figure 2. (A) Gating strategy for the identification of MSC subtypes (for Figure 2A). **(B)** Representative flow cytometry plots showing *Il15*^{GFP} expression in Tomato^{+} and Tomato^{-} BM stromal cells (Lin^{-}CD45^{-}CD31^{-}) in *Il15*^{GFP} single reporter mice (top), *Prx1-Cre* Tomato single reporter mice (middle) and *Il15*^{GFP} *Prx1-Cre* Tomato double reporter mice (bottom). **(C)** Gating strategy for (pre)hypertrophic and proliferating chondrocytes (Lin: CD71/TER-119/CD31/CD19), for Figure 2C. **(D)** Gating strategy for arteriolar and sinusoidal ECs (for Figure 2D). **(E)** qPCR quantitation showing the log2 fold change of the indicated transcripts in *Il15*$^{GFP-}$ versus *Il15*$^{GFP+}$ sorted stromal cells (pre-enriched for CD73^{+} and gated on Lin^{-}CD45^{-}CD31^{-}GFP$^{+/-}$) compared to cDNA isolated from total bone/BM. n.d., not detected. **(F)** qPCR quantitation (normalized to bAct) of the indicated markers in *Il15*$^{GFP-}$ versus *Il15*$^{GFP+}$ sorted stromal cells (pre-enriched for CD73^{+} and gated on Lin^{-}CD45^{-}CD31^{-}GFP$^{+/-}$). Bar graphs show mean ± SEM with each dot representing a biological replicate (n= 2-4 from 2 independent experiments). *P* values indicated are from unpaired two-tailed Student's t-tests. * $P < 0.05$, ** $P < 0.01$.

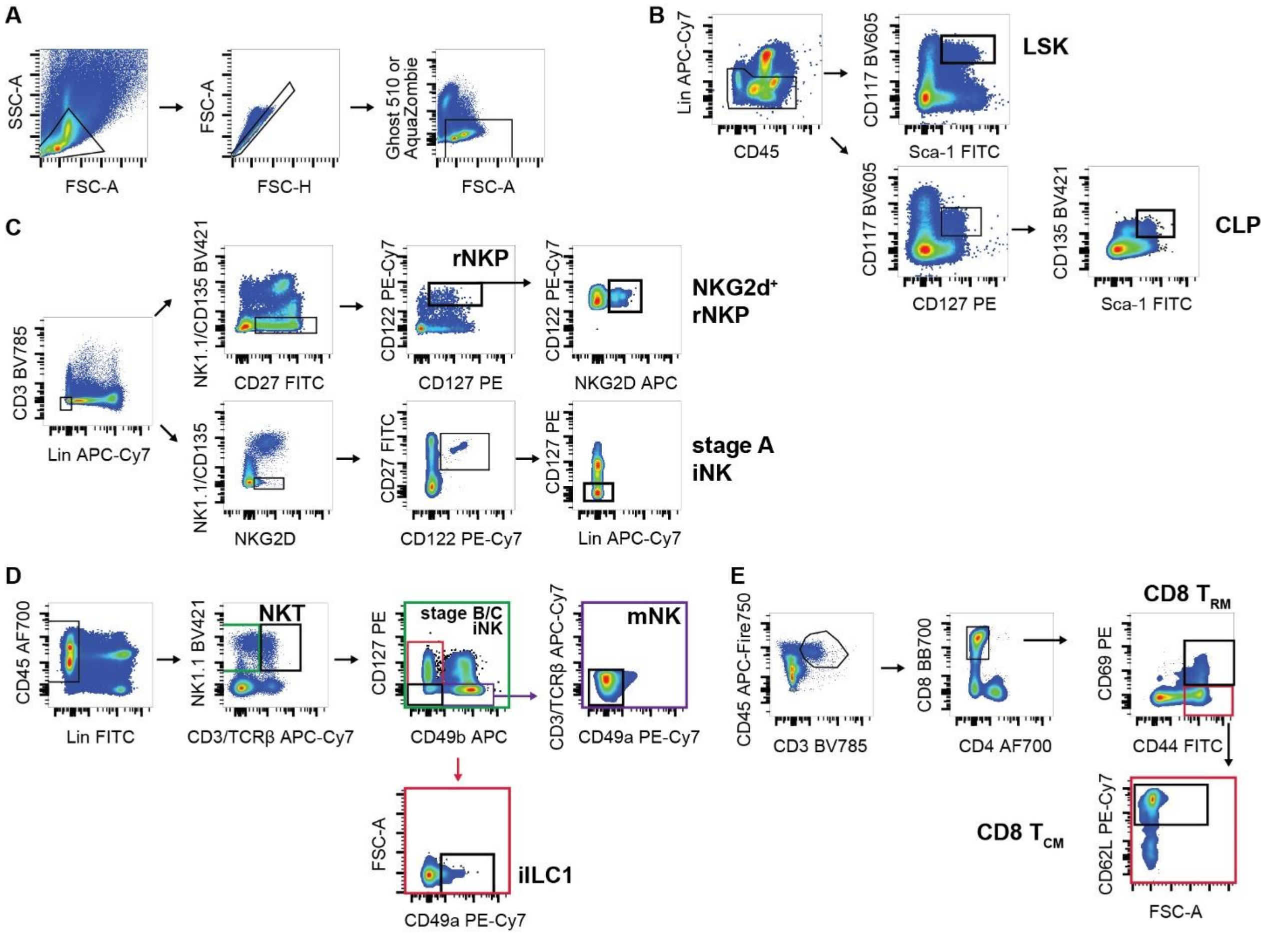

Supplementary Figure 3. Flow cytometry gating strategies to identify immune cell lineages, related to Figure 3. (A) Common quality control gating used in all flow cytometry panels before marker-specific identification of cell populations. **(B)** Gating strategy for identifying LSK (Lin$^-$Sca-1$^+$cKit$^+$) and CLP (Lin$^-$cKit$^+$CD127$^+$Sca-1$^+$CD135$^+$). Lin: CD3/CD19/Gr-1/NK1.1/TER-119 (for Figure 3B and Suppl. Figure 6C). **(C)** Gating strategy for rNKP (Lin$^-$CD3$^-$NK1.1$^-$CD135$^-$CD27$^+$CD122$^+$CD127$^+$ and, if indicated NKG2D$^+$) and stage A iNK (Lin$^-$CD3$^-$NK1.1$^-$CD135$^-$CD27$^+$CD122$^+$CD127$^-$). Lin: CD19/Gr-1/TER-119/CD11c (for Figures 3B, 4A, 5B and Suppl. Figures 8C and 9A). **(D)** Gating strategy for NKT cells (CD45$^+$Lin$^-$NK1.1$^+$CD3$^+$), iILC1 (CD45$^+$Lin$^-$NK1.1$^+$CD3$^-$CD127$^+$CD49b$^-$CD49a$^+$) and CD49b$^+$ mNK cells (CD45$^+$Lin-NK1.1$^+$CD3$^-$CD49b$^+$CD127$^-$CD49a$^-$), for Figures 3B, 3E, 4A, 4D, 5B and Suppl. Figures 4C-D, 5A, 6B-E, 8C-G, 9B). **(E)** Gating strategy for CD8$^+$ T_{CM} (CD45^{hi}CD3$^+$CD8$^+$CD4$^-$CD44$^+$CD69$^-$CD62L$^+$) and CD8$^+$ T_{RM} cells (CD45^{hi}CD3$^+$CD8$^+$CD4$^-$CD44$^+$CD69$^+$), for Figures 3E, 4D, 5B-F and Suppl. Figures 4A-D, 6B-E, 8A, 8E-G and 9B.

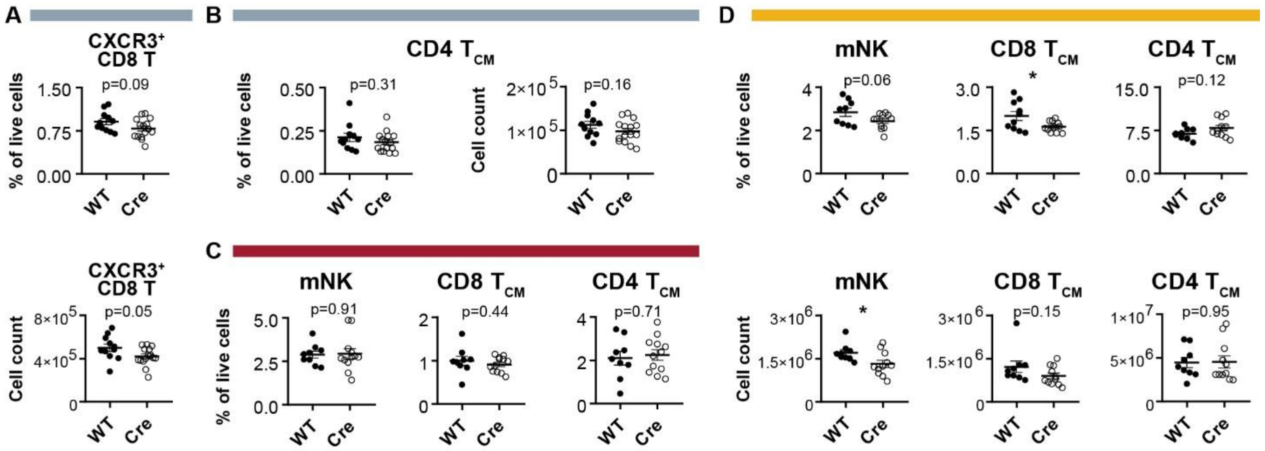

Supplementary Figure 4. Frequency of immune cell subsets in the BM, spleen and blood of *Il15^flox/flox^ Prx1-Cre* mice, related to Figure 3. (A) Relative abundance (top) and absolute numbers (bottom) of CXCR3$^+$ CD8$^+$ T cells in the BM (grey bars) of *Il15*$^{flox/flox}$ *Prx1-Cre* and WT and littermates (n= 11-15 from 4 independent experiments). **(B)** CD4$^+$ T_{CM} cells (relative abundance and absolute nrs) in the BM of *Il15*$^{flox/flox}$ *Prx1-Cre* and WT and littermates (n= 11-15 from 4 independent experiments). **(C)** mNK cells, CD8$^+$ T_{CM} and CD4$^+$ T_{CM} abundance in the blood (red bar) of *Il15*$^{flox/flox}$ *Prx1-Cre* and WT littermates (n= 9-12 from 3 independent experiments). **(D)** Relative abundance (top) and total numbers (bottom) of mNK cells, CD8$^+$ T_{CM} and CD4$^+$ T_{CM} in the spleen (yellow bar) of *Il15*$^{flox/flox}$ *Prx1-Cre* and WT littermates. (n= 10-11 from 3 independent experiments). Dot plots show mean ± SEM with each dot representing a biological replicate. Numbers above the dot plots indicate *P* values from unpaired two-tailed Student's t-tests. * $P < 0.05$.

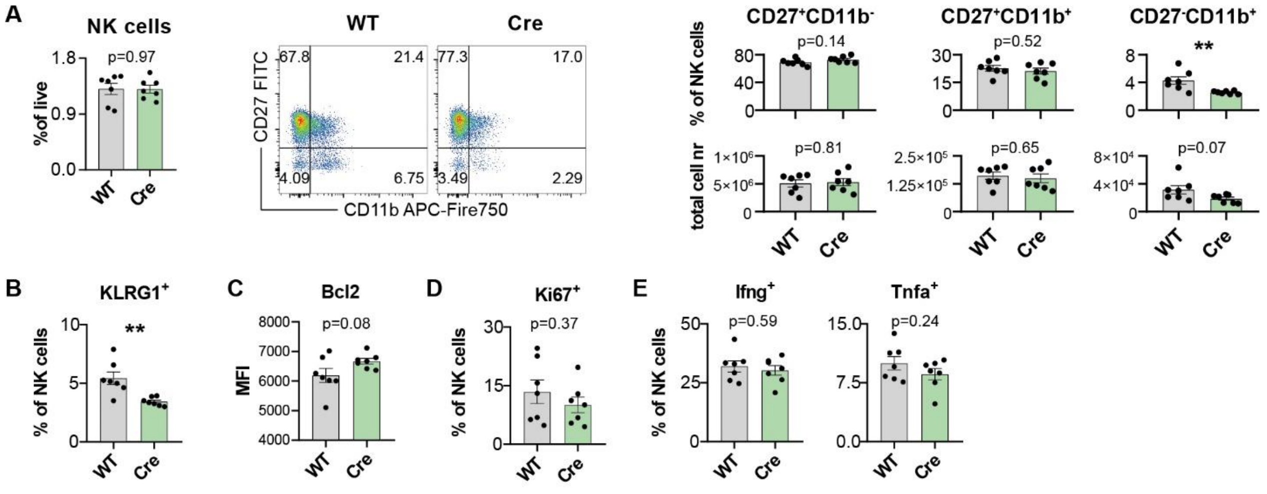

Supplementary Figure 5. Evaluation of functional and maturation markers on BM NK cells in *Il15$^{flox/flox}$ Prx1-Cre* mice, related to Figure 3. (A) Relative quantification of CD27 and CD11b expression on NK cell subsets from 20-30 week old *Il15$^{flox/flox}$ Prx1-Cre* mice and WT littermates. Abundance of total NK1.1$^+$CD3$^-$ NK cells (left) and relative quantification of CD11b/CD27 subsets among total BM NK cells (right). Representative CD11b/CD27 flow cytometry plots of NK1.1$^+$CD3$^-$ NK cells are shown in the middle (n=7 per group from 2 independent experiments). **(B)** Relative quantification of KLRG1-expressing NK cells in the BM of *Il15$^{flox/flox}$ Prx1-Cre* mice and WT littermates (n=7 per group from 2 independent experiments). **(C)** Mean fluorescence intensity (MFI) of Bcl2 in BM NK cells of *Il15$^{flox/flox}$ Prx1-Cre* mice and WT littermates(n=7 per group from 2 independent experiments). **(D)** Relative quantification of Ki-67$^+$ proliferating NK cells in the BM of *Il15$^{flox/flox}$ Prx1-Cre* mice and WT littermates (n=7 per group from 2 independent experiments). **(E)** Relative quantification of IFN-γ and TNF-α expressing NK cells after 3 hours *in vitro* activation with PMA/ionomycin of BM extracts from *Il15$^{flox/flox}$ Prx1-Cre* mice and WT littermates (n=7 per group from 2 independent experiments). Bar graphs show mean ± SEM with each dot representing a biological replicate. Numbers indicate *P* values from unpaired two-tailed Student's t-tests. ** $P < 0.01$.

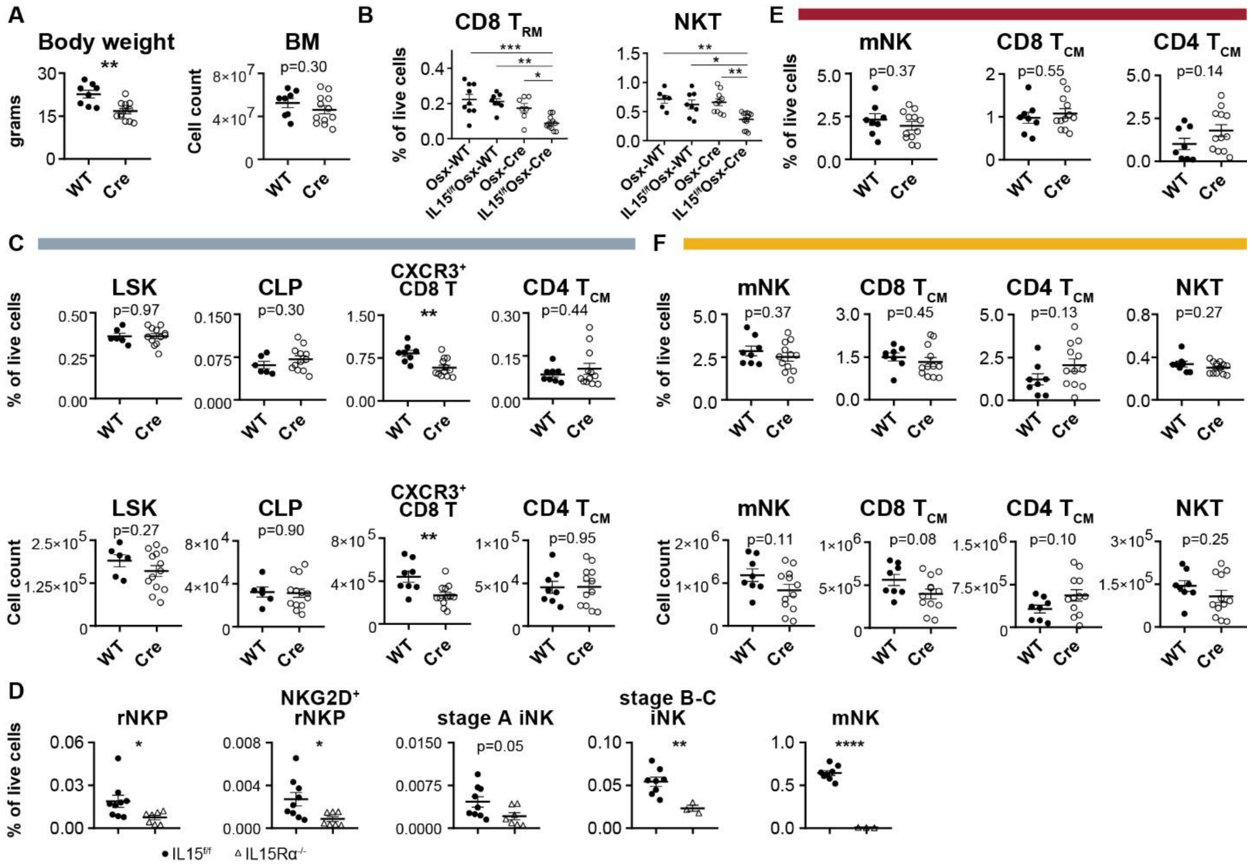

Supplementary Figure 6. Frequency of immune cell subsets in the BM, spleen and blood of *Il15*$^{flox/flox}$ *Osx-Cre* mice, related to Figure 4. (A) Growth and bone marrow cellularity parameters of *Il15*$^{flox/flox}$ and *Il15*$^{flox/flox}$ *Osx-Cre* littermates (n=8-12 from 5 independent experiments). **(B)** Abundance of $CD8^+$ T_{RM} and NKT cells in the BM of C57BL/6J (WT), *Il15*$^{flox/flox}$ *Osx-Cre* littermates as well as Cre only control mice (*Osx-Cre*). Asterisks indicate significant differences from Tukey's multiple comparisons test post one-way ANOVA (n=7-11). **(C)** Relative abundance and cell numbers of LSK, CLP, $CXCR3^+$ $CD8^+$ T cells and $CD4^+$ T_{CM} in the BM (grey bar) of *Il15*$^{flox/flox}$ and *Il15*$^{flox/flox}$ *Osx-Cre* littermates (n=6-12 from 4 independent experiments). **(D)** Relative abundance of rNKP, rNKP NKG2D+, stage A, B/C iNK and mNK in the BM of *Il15*$^{flox/flox}$ and *Il15rα*$^{-/-}$ mice (n=3-9 from 3-4 independent experiments). **(E)** Abundance of $CD49b^+$ mNK cells, $CD8^+$ T_{CM} and $CD4^+$ T_{CM} in the blood (red bar) of *Il15*$^{flox/flox}$ and *Il15*$^{flox/flox}$ *Osx-Cre* littermates (n=8-12 from 5 independent experiments). **(F)** Shown is the relative abundance and total number of $CD49b^+$ mNK cells, $CD8^+$ T_{CM} and $CD4^+$ T_{CM} in the spleen (yellow bar) of *Il15*$^{flox/flox}$ and *Il15*$^{flox/flox}$ *Osx-Cre* littermates (n=8-12 from 5 independent experiments). Dot plots show mean ± SEM with each dot representing a biological replicate. Numbers indicate *P* values from unpaired two-tailed Student's t-tests. * $P < 0.05$, ** $P < 0.01$, *** $P < 0.001$, **** $P < 0.0001$. Source data are provided as a Source Data file.

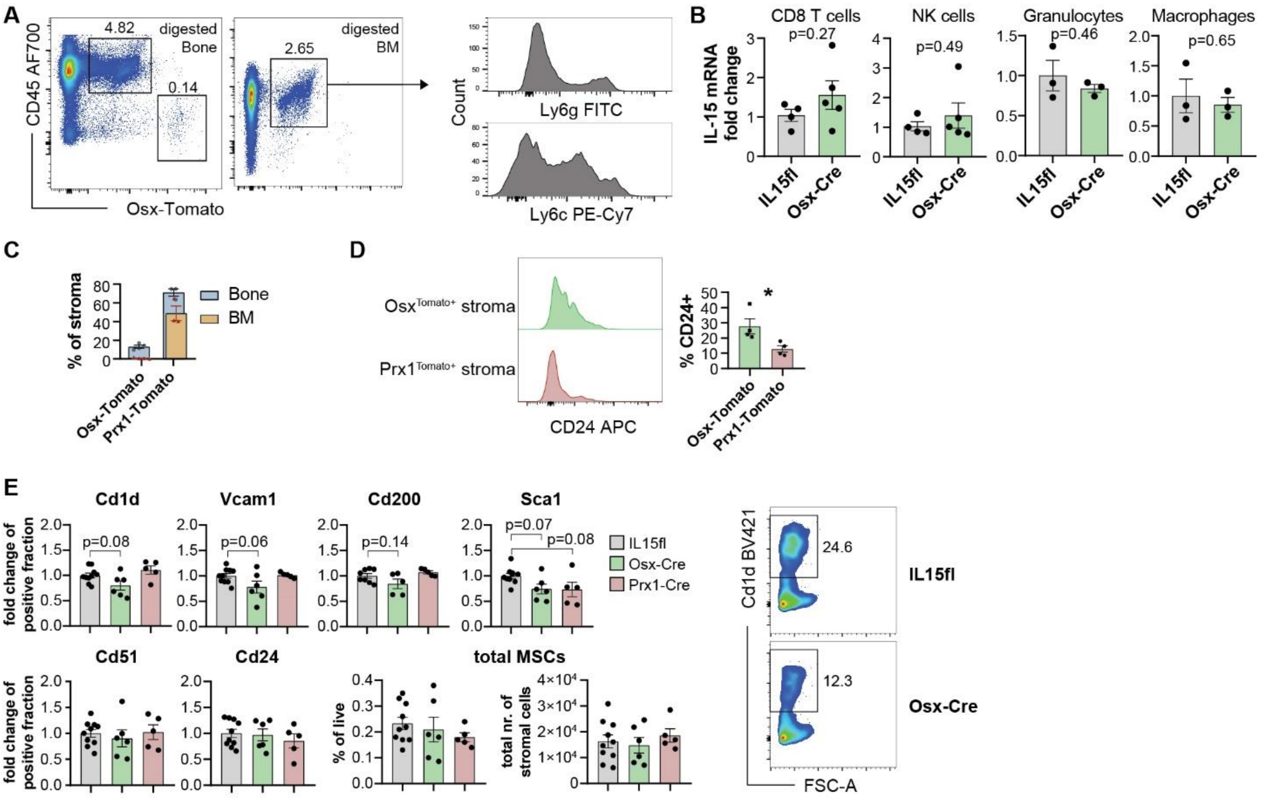

Supplementary Figure 7. Characterization of *Osx-Cre* and *Prx1-Cre* targeted cells and stromal cell landscape. (A) Representative flow cytometry plots showing CD45$^-$ and CD45$^+$ cells from digested bone and BM of Osx-Tomato mice. Histograms show Ly6G and Ly6C expression of CD45$^+$Tomato^{dim} cells in BM. **(B)** qPCR quantification of *Il15* mRNA in purified CD8$^+$ T cells, NK cells, SSC^{hi} Ly6G$^+$ granulocytes or monocytes/macrophages of *Il15$^{flox/flox}$* mice and *Il15$^{flox/flox}$ Osx-Cre* littermates (n=3-5 biological replicates from 2 independent experiments; *P* values from unpaired two-tailed Student's t-tests). **(C)** Comparison of relative abundance of Tomato$^+$ cells in BM and bone of Osx-Tomato and Prx1-Tomato mice among total (CD45$^-$Lin$^-$CD31$^-$) stromal cells (n=3-4 biological replicates per group). **(D)** Relative abundance of CD24$^+$ cells among total Tomato$^+$ cells in digested bone of Osx-Tomato and Prx1-Tomato mice (n=4 biological replicates per group from 2 independent experiments; asteristk indicates p value < 0.05 from an unpaired two-tailed Student's t-test). **(E)** Normalized abundance of cells positive for the indicated marker among total stroma compared to *Il15$^{flox/flox}$* controls. P values from Dunnett's multiple comparison test post one-way ANOVA which are below 0.2 are indicated above the plots (left). Right: Representative flow cytometry plot of CD1d expression in stromal cells of *Il15$^{flox/flox}$* mice and *Il15$^{flox/flox}$ Osx-Cre* littermates (n=5-10 biological replicates per group). Source data are provided as a Source Data file.

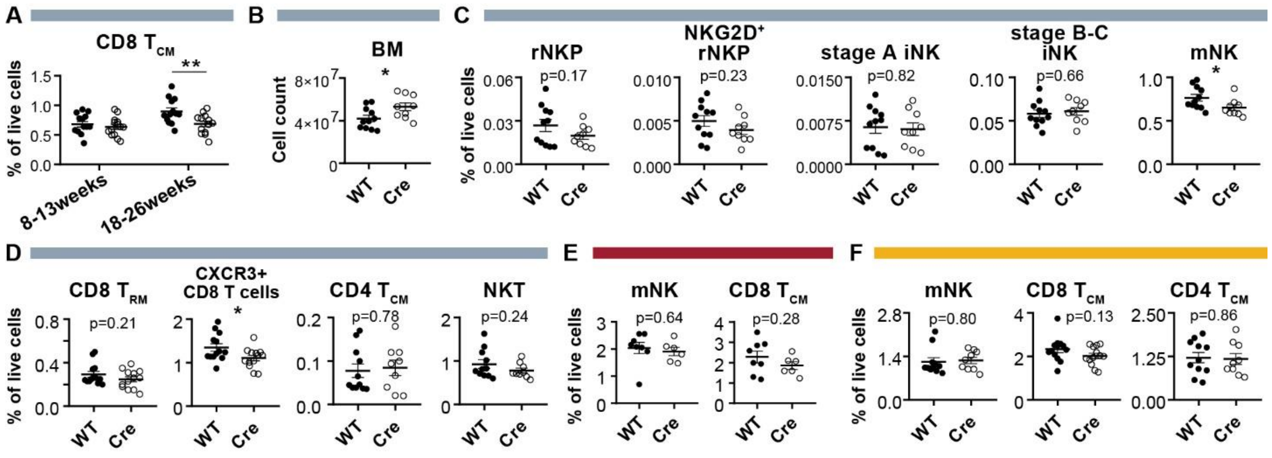

Supplementary Figure 8. Frequency of immune cell subsets in *Il15$^{flox/flox}$ Lepr-Cre* mice, related to Figure 4. (A) Frequency of CD8^{+} T$_{CM}$ cells in 8-13 and 18-26 week old *Il15$^{flox/flox}$* (WT) and *Il15$^{flox/flox}$ Lepr-Cre* (Cre) mice. Asterisks indicate significant differences from Sidak's multiple comparisons test after two-way ANOVA (with genotype and age as significantly different factors; n=13-14 per group). **(B)** Increased BM cellularity in 18-26 week old *Lepr-Cre* mice. **(C)** BM relative abundance of rNKP, NKG2D^{+} rNKP, stage A and B/C iNK cells and CD49b^{+} mNK cells in the BM of 18-26 week old *Lepr-Cre* mice (n=9-11 from 3 independent experiments). **(D)** Relative abundance of CD8^{+} T$_{RM}$ cells, CXCR3^{+} CD8^{+} T cells, CD4^{+} T$_{CM}$ and NKT cells in the BM of 18-26 week old *Lepr-Cre* mice (n=13 per group from 4 independent experiments). **(E)** Relative abundance of CD8^{+} T$_{CM}$ and mNK cells in the blood (red bar) of 18-26 week old *Lepr-Cre* mice (n=6-8 from 2 independent experiments). **(F)** Relative abundance of mNK, CD8^{+} T$_{CM}$ and CD4^{+} T$_{CM}$ cells in the spleen (yellow bar) of 18-26 week old *Lepr-Cre* mice (n=9-13 from 3-4 independent experiments). Dot plots show mean ± SEM with each dot representing a biological replicate. Numbers indicate *P* values from unpaired two-tailed Student's t-tests. * $P < 0.05$, ** $P < 0.01$. Source data are provided as a Source Data file.

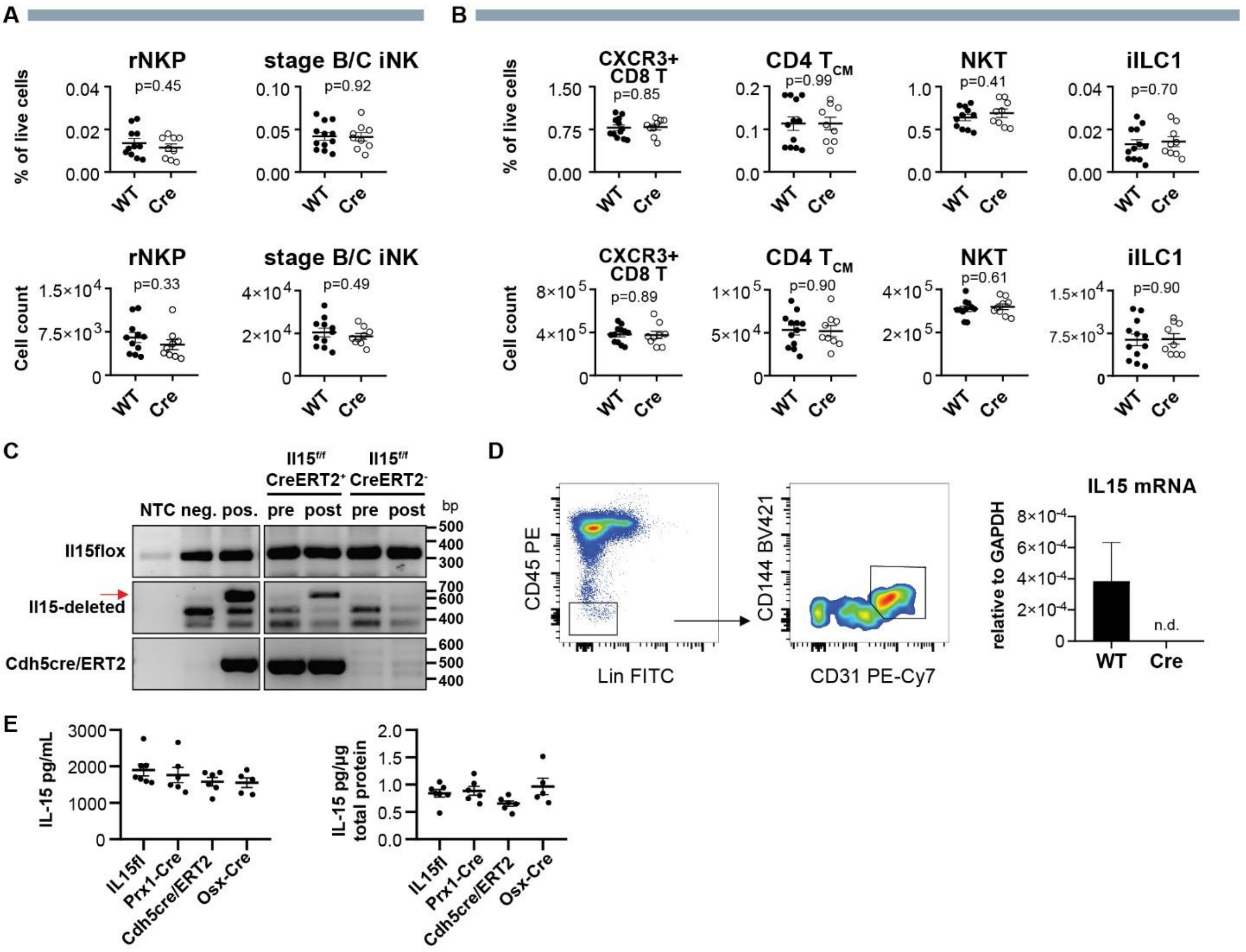

Supplementary Figure 9 Frequency of immune cell subsets in *Il15^flox/flox^ Cdh5-Cre^ERT2^* mice, related to Figure 5. (A) Relative abundance (top) and absolute numbers (bottom) of rNKP and stage B/C iNK cells in the BM of tamoxifen-treated *Il15*$^{flox/flox}$ (WT) and *Il15*$^{flox/flox}$ *Cdh5-Cre*^{ERT2} (Cre) littermates (n=9-11 from 3 independent experiments). **(B)** Relative abundance (top) and absolute numbers (bottom) of CXCR3^{+} CD8^{+} T cells, CD4^{+} T_{CM} cells, NKT and iILC1 cells in the BM of tamoxifen-treated mice (n=9-12 from 3 independent experiments). **(C)** Genotyping of toe/ear clips of *Il15*$^{flox/flox}$ *Cdh5-Cre*^{ERT2} mice before and after Tamoxifen induction showing a detectable IL-15 deletion. NTC = no template control; neg: negative (*Il15*$^{flox/flox}$) control; pos: positive (Cre^{+} IL15-deleted) control. **(D)** Gating strategy for sorted endothelial cells from spleens of tamoxifen-induced *Il15*$^{flox/flox}$ (WT) and *Il15*$^{flox/flox}$ *Cdh5-Cre*^{ERT2} (Cre) littermates (left) and qPCR quantification of *Il15* mRNA. **(E)** IL-15 ELISA from BM lysates of the indicated mouse strains, concentration (left) and normalized to total protein quantification (right); n=5-7 per group from 2 independent experiments. One-way ANOVA yielded p values of 0.38 and 0.12, respectively. Dot plots show mean ± SEM with each dot representing a biological replicate. Numbers indicate *P* values from unpaired two-tailed Student's t-tests. Source data are provided as a Source Data file.

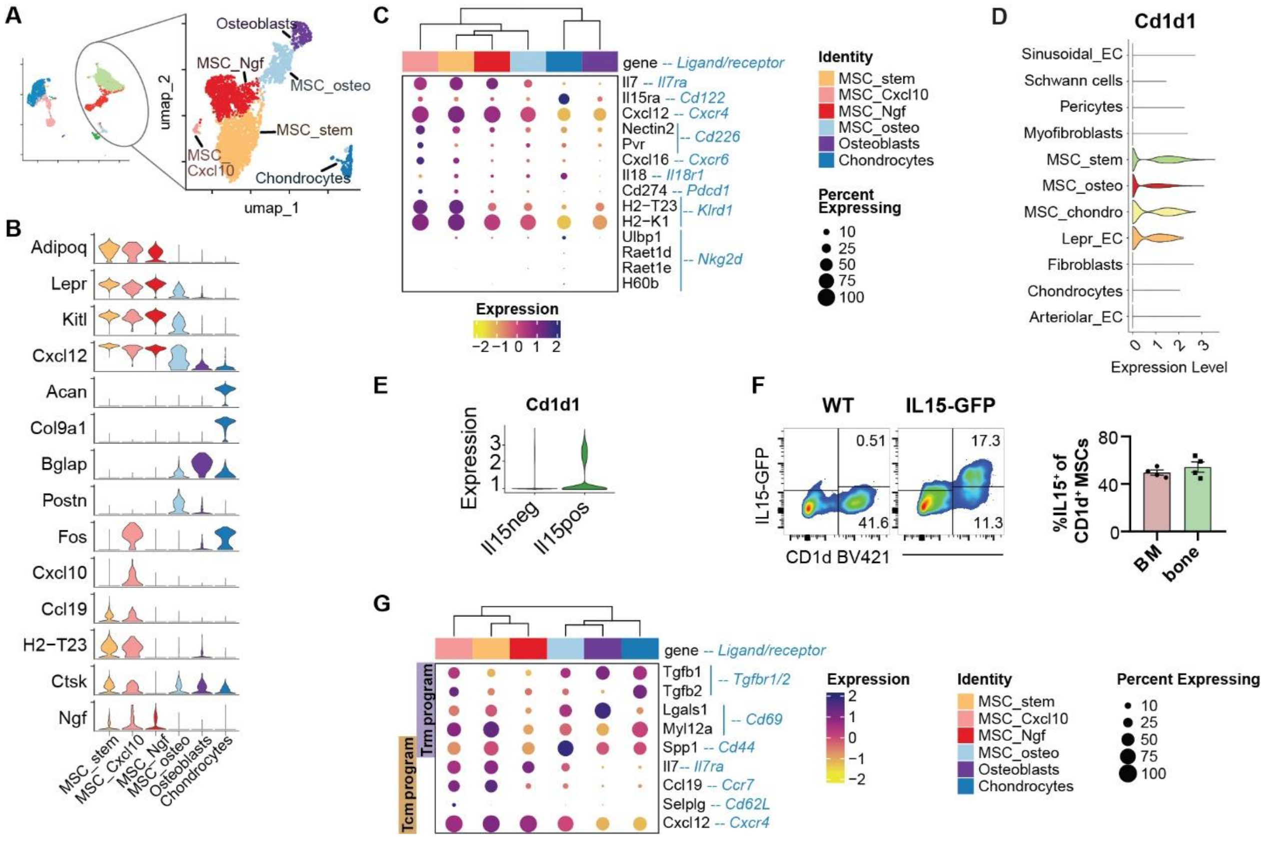

Supplementary Figure 10. Differential expression of receptors and ligands among *Il15*$^{GFP+}$ MSC subsets, related to Figures 1, 3 and 4. (A) The *Il15*$^{GFP+}$ scRNA-seq dataset shown in Figure 1 was used for unsupervised subclustering of MSC and chondrocyte subsets, identifying four distinct MSC transcriptional states. **(B)** Violin plots showing cluster-defining marker expression for MSC and osteoblast/chondrocyte subclusters revealing an inflammatory MSC_Cxcl10 subset, a *Ccl19*$^{+}$ MSC_stem subset and an MHC-I^{low} Ngf^{high} MSC_Ngf subset. **(C)** Dot plot of *Il15*$^{GFP+}$ MSCs showing k-means clustering of cell types by expression of the indicated ligands/receptors relevant for NK cell precursors. **(D)** Violin plot showing expression of *Cd1d1* in clusters of the public scRNA-seq dataset shown in Figure 1. **(E)** Violin plot showing *Cd1d1* expression in *Il15*$^{-}$ versus *Il15*$^{+}$ cells of the public scRNA-seq dataset. **(F)** Representative flow cytometry plot (left) of CD1d and *Il15*^{GFP} coexpression in Lin^{-}CD31^{-}CD45^{-} cells of BM and bone (right) from WT and *Il15*^{GFP} mice. Bar graphs show mean ± SEM. **(G)** Dot plot of *Il15*$^{GFP+}$ MSCs showing k-means clustering of cell types by expression of the indicated ligands/receptors relevant for CD8^{+} T$_{RM}$ versus CD8^{+} T$_{CM}$ cells. The respective ligands/receptors on memory CD8^{+} T cell subsets are highlighted in blue.

Supplementary Tables

Supplementary Table 1. Genotyping primers

Primer name	Forward	Reverse
Cdh5-Cre/ERT2	TCCTGATGGTGCCTATCCTC	CCTGTTTTGCACGTTCACCG
Cxcl12^{DsRed} KI	ACGGCACCTTCATCTACCAC	ACCACGGTGTAGTCCTCGTT
Cxcl12^{DsRed} WT	CTGGTTTTCGCCTCTAAAGC	CAGAGCTGCGAGCCTTTC
Il15 flox	AGAGCCTTGCCACTAGCTACA	AGAAGGCTTTGCAATGTTCA
Il15 flox deleted	AGGATGCAGGAGATGTTTGG	CCTCTGCACCTTGACTGGTT
Il15^{GFP} KI	ACTTCAAGATCCGCCACAAC	CACACACAGCCAAACACACA
Il15^{GFP} WT	GCCACTTGGATCACATGAAA	CACACACAGCCAAACACACA
Lepr-Cre	GCTGGAAGATGGCGATTAGC	TCTTCTTTCCAGAGTTCAGATGT
Lepr-WT	CCCAATTTCAAACCTGTTCC	TCTTCTTTCCAGAGTTCAGATGT
Osx-Cre	GAGAATAGGAACTTCGGAATAGTAAC	CCCTGGAAGTGACTAGCATTG
Osx-WT	AGAGAGCTCCCCTCAATTATGT	AGCCACTTCTAGCACAAAGAACT
Prx1-Cre	GCGGTCTGGCAGTAAAAACTATC	GTGAAACAGCATTGCTGTCACTT
Prx1-WT	CTAGGCCACAGAATTGAAAGATCT	GTAGGTGGAAATTCTAGCATCATCC
Rosa26 tdTomato	AGGGCGAGGAGCTGTTCA	TGAAGTCGATGCCCTTCAG
Rosa26 WT	CTGGCTTCTGAGGACCG	CAGGACAACGCCCACACA

Supplementary Table 2. qPCR primers

Primer name	Forward	Reverse
Adipoq	AGATGGCACTCCTGGAGAGAAG	ACATAAGCGGCTTCTCCAGGCT
bAct	GCTCTTTTCCAGCCTTCCTT	CTTCTGCATCCTGTCAGCAA
Bmp6	CTTTCCTCAACGACGCGGACAT	CCTCAGGAATCTGGGATAGGTTG
Cd146	CGAGGCAGAAAGTAACCAGGAC	GTCTCACGTTGTTTAGCTGGAGG
Cd200	CTCTCCACCTACAGCCTGATT	AGAACATCGTAAGGATGCAGTTG
Cd44	AATGGCTCATCATCTTGGCA	GCTCACTGGGTTTCCTGTCT
Cd51	GTGTGAGGAACTGGTCGCCTAT	CCGTTCTCTGGTCCAACCGATA
Ctsk	CTTCCAATACGTGCAGCAGA	TCTTCAGGGCTTTCTCGTTC
Cxcl9	CCTAGTGATAAGGAATGCACGATG	CTAGGCAGGTTTGATCTCCGTTC
Ddr2	TCATCCTGTGGAGGCAGTTCTG	CTGTTCACTTGGTGATGAGGAGC
Gapdh	GTGGACCTCATGGCCTACAT	TGTGAGGGAGATGCTCAGTG
Il15	GTGACTTTCATCCCAGTTGC	GCAAGGTAGAGCACGTTTC
Runx2	CGTGGCCTTCAAGGTTGTA	GCCCACAAATCTCAGATCGT
Sca1	CCTACCCTGATGGAGTCTGTGT	CACGTTGACCTTAGTACCCAGG
Sp7	GCCTACTTACCCATCTGACTTT	GCCCACTATTGCCAACCGC

Supplementary Table 3. Top 10 differentially expressed genes in each of the 11 scRNA-seq clusters (related to Figure 1A)

	P_val	Avg_log2 FC	Pct.1	Pct.2	P_val_adj	Cluster	Gene
1	2,8E-285	5,193864919	0,979	0,397	7,6739E-281	Fibroblasts	Dcn
2	2,5E-238	3,114930377	0,969	0,42	6,786E-234	Fibroblasts	S100a6
3	9,4E-236	4,527045169	0,926	0,319	2,533E-231	Fibroblasts	Gsn
4	1E-226	3,402532762	0,894	0,262	2,7131E-222	Fibroblasts	Comp
5	8,1E-218	4,784299769	0,77	0,102	2,1949E-213	Fibroblasts	Sod3
6	2,8E-217	5,918030116	0,791	0,13	7,4839E-213	Fibroblasts	Prg4
7	2,8E-215	5,86210151	0,76	0,07	7,541E-211	Fibroblasts	Igfbp6
8	6,1E-214	6,202107068	0,705	0,042	1,6586E-209	Fibroblasts	Cilp2
9	2,3E-213	4,625561672	0,795	0,111	6,2284E-209	Fibroblasts	Nbl1
10	2,6E-205	2,18528366	0,984	0,726	7,1484E-201	Fibroblasts	Cd9
11	0	6,020407708	0,919	0,044	0	Sinusoidal_EC	Stab2
12	0	4,90500337	0,984	0,153	0	Sinusoidal_EC	Tfpi
13	1,2E-307	4,538616072	0,97	0,135	3,3441E-303	Sinusoidal_EC	Lrg1
14	1,1E-300	3,919179497	0,995	0,366	3,1049E-296	Sinusoidal_EC	Fabp4
15	8,6E-280	2,573164532	0,997	0,76	2,3183E-275	Sinusoidal_EC	Sepp1
16	1,5E-272	3,431622534	0,952	0,139	3,9288E-268	Sinusoidal_EC	Cldn5
17	1,1E-270	3,679610801	0,949	0,355	3,0935E-266	Sinusoidal_EC	Il6st
18	2,3E-265	4,50337456	0,868	0,108	6,2446E-261	Sinusoidal_EC	Gm1673
19	4,5E-265	5,606065393	0,835	0,034	1,2278E-260	Sinusoidal_EC	Gpm6a
20	1,7E-263	3,232338153	0,946	0,324	4,5906E-259	Sinusoidal_EC	Mafb
21	0	5,355327314	0,95	0,071	0	MSC_stem	Esm1
22	0	5,941845713	0,958	0,089	0	MSC_stem	Adipoq
23	0	5,403938479	0,989	0,196	0	MSC_stem	Hp
24	0	4,800590315	1	0,466	0	MSC_stem	Cxcl12
25	8,9E-307	4,719903699	0,963	0,232	2,41E-302	MSC_stem	Lpl
26	5,4E-305	3,864456402	0,984	0,392	1,4494E-300	MSC_stem	Gas6
27	1,3E-297	3,66139296	0,979	0,289	3,3937E-293	MSC_stem	Serping1
28	5,6E-284	3,588607891	0,969	0,244	1,5135E-279	MSC_stem	Cxcl14
29	2,6E-281	5,189158792	0,871	0,059	6,9069E-277	MSC_stem	Dpep1
30	1,7E-274	4,862092892	0,885	0,08	4,5103E-270	MSC_stem	Gdpd2
31	0	4,85403737	0,954	0,195	0	Chondrocytes	Col9a2
32	0	4,808543521	0,954	0,198	0	Chondrocytes	Hapln1
33	8,8E-307	4,842346105	0,958	0,228	2,3905E-302	Chondrocytes	Col9a3
34	1,2E-305	4,53023849	0,96	0,249	3,1306E-301	Chondrocytes	3110079O15Rik
35	3,3E-303	4,696818388	0,95	0,179	9,0436E-299	Chondrocytes	Acan
36	5,5E-303	5,773857146	0,917	0,108	1,4881E-298	Chondrocytes	Matn3
37	3,5E-301	4,850621022	0,944	0,16	9,5438E-297	Chondrocytes	Col9a1
38	2,9E-299	4,339866331	0,953	0,205	7,8745E-295	Chondrocytes	Col11a2
39	4,2E-292	4,366669885	0,937	0,2	1,1238E-287	Chondrocytes	Col27a1
40	5,9E-289	4,363098372	0,938	0,164	1,5949E-284	Chondrocytes	Lect1
41	2,4E-246	3,907515442	0,905	0,239	6,5562E-242	Arteriolar_EC	Ly6c1
42	1E-214	2,419220785	0,944	0,302	2,728E-210	Arteriolar_EC	Egfl7
43	5,4E-201	2,927178081	0,864	0,25	1,4641E-196	Arteriolar_EC	Pecam1
44	1,2E-196	3,799341538	0,714	0,077	3,1232E-192	Arteriolar_EC	Cd34
45	1,7E-195	2,048382385	0,954	0,508	4,4716E-191	Arteriolar_EC	Ly6e
46	3,4E-179	2,991400709	0,869	0,305	9,2069E-175	Arteriolar_EC	Ly6a
47	2,1E-178	2,817600024	0,894	0,506	5,5876E-174	Arteriolar_EC	Tm4sf1
48	5,2E-169	3,576409291	0,755	0,231	1,4E-164	Arteriolar_EC	Slc9a3r2
49	6,8E-158	2,825947104	0,751	0,193	1,8475E-153	Arteriolar_EC	Ecscr
50	1,1E-154	2,305331578	0,824	0,385	2,9269E-150	Arteriolar_EC	Ramp2
51	3,7E-210	1,777434744	0,992	0,839	9,8681E-206	MSC_osteo	Cd63
52	1,1E-209	2,805860996	0,929	0,309	3,0127E-205	MSC_osteo	Cfh
53	1,9E-199	4,258652642	0,956	0,565	5,1244E-195	MSC_osteo	Spp1
54	9,6E-195	3,710231325	0,821	0,152	2,5938E-190	MSC_osteo	Alpl
55	7E-184	3,039132176	0,835	0,28	1,8926E-179	MSC_osteo	Tnc
56	8,3E-176	2,830918553	0,812	0,214	2,2319E-171	MSC_osteo	Fap
57	3,8E-166	2,560842512	0,872	0,297	1,0334E-161	MSC_osteo	Olfml3
58	2,6E-162	6,784646751	0,728	0,147	7,0585E-158	MSC_osteo	Bglap2
59	1,1E-161	4,988121838	0,592	0,027	3,015E-157	MSC_osteo	Slc36a2

60	6,8E-160	2,132450252	0,829	0,264	1,8328E-155	MSC_osteo	Pth1r
61	2,63E-54	1,428115961	0,776	0,24	7,11505E-50	Lepr_EC	Pappa
62	3,91E-42	1,388670343	0,683	0,23	1,0572E-37	Lepr_EC	Ebf3
63	1,02E-41	1,047995132	0,822	0,284	2,76212E-37	Lepr_EC	Lepr
64	5,73E-35	1,086266289	0,73	0,262	1,54935E-30	Lepr_EC	Chrdl1
65	3,23E-33	1,075653365	0,649	0,236	8,72552E-29	Lepr_EC	Cyp1b1
66	4,93E-26	1,15865845	0,517	0,183	1,33329E-21	Lepr_EC	Grem1
67	2,22E-21	1,236106683	0,402	0,123	6,00444E-17	Lepr_EC	Slc26a7
68	2,61E-21	1,06973896	0,494	0,185	7,06653E-17	Lepr_EC	Mme
69	2,96E-21	1,146559337	0,432	0,165	7,99549E-17	Lepr_EC	Plxna2
70	6,94E-19	1,253297997	0,251	0,068	1,87499E-14	Lepr_EC	Cbln1
71	1,2E-180	5,789938759	0,667	0,015	3,2392E-176	Myofibroblasts	Chodl
72	4,7E-179	1,546055298	0,99	0,97	1,2751E-174	Myofibroblasts	Rps5
73	1,8E-172	5,331437104	0,678	0,043	4,9483E-168	Myofibroblasts	Des
74	2,1E-172	1,390434012	0,998	0,984	5,6875E-168	Myofibroblasts	Rps19
75	9,9E-170	1,388857183	0,995	0,983	2,6836E-165	Myofibroblasts	Rpl18a
76	2,9E-167	1,241766919	0,997	0,989	7,8148E-163	Myofibroblasts	Rpl13
77	2E-164	1,55793989	0,991	0,949	5,3358E-160	Myofibroblasts	Rps6
78	6,3E-154	2,513837614	0,922	0,599	1,69E-149	Myofibroblasts	Gm8730
79	5,2E-151	1,435384193	0,988	0,95	1,3926E-146	Myofibroblasts	Rpl3
80	1,9E-148	6,063421896	0,584	0,03	5,0272E-144	Myofibroblasts	Crlf1
81	4,6E-223	12,77534593	0,972	0,01	1,2417E-218	Schwann cells	Plp1
82	1,3E-191	10,88710023	0,874	0,011	3,4789E-187	Schwann cells	Mbp
83	6,6E-175	7,043927494	0,797	0,015	1,771E-170	Schwann cells	Aspa
84	1,1E-169	7,141947448	0,804	0,018	3,0561E-165	Schwann cells	Plekhb1
85	4,4E-169	8,796868376	0,776	0,011	1,1895E-164	Schwann cells	Mal
86	1,5E-155	9,246320366	0,657	0,002	4,1403E-151	Schwann cells	Cadm4
87	3,7E-153	6,938459255	0,818	0,031	1,0104E-148	Schwann cells	Cmtm5
88	1,4E-152	9,724287808	0,664	0,002	3,7883E-148	Schwann cells	Sox10
89	1,1E-150	9,250014737	0,657	0,004	2,9069E-146	Schwann cells	Pllp
90	1,6E-147	10,87109227	0,65	0,004	4,3957E-143	Schwann cells	Mag
91	2,8E-277	7,96777838	0,859	0,077	7,4461E-273	Pericytes	Acta2
92	2,3E-272	7,513549444	0,838	0,052	6,3109E-268	Pericytes	Tagln
93	1,2E-270	5,611410286	0,897	0,198	3,2722E-266	Pericytes	Myl9
94	1,1E-267	5,626539045	0,898	0,184	2,9649E-263	Pericytes	Tpm2
95	1,2E-247	4,436251805	0,866	0,175	3,2685E-243	Pericytes	Mustn1
96	1,4E-245	4,142748512	0,868	0,129	3,6609E-241	Pericytes	Sparcl1
97	2,5E-243	8,552837662	0,795	0,048	6,6832E-239	Pericytes	Rgs5
98	6,6E-239	8,558970952	0,761	0,013	1,7924E-234	Pericytes	Myh11
99	5,3E-231	4,199578709	0,809	0,112	1,4224E-226	Pericytes	Notch3
100	1,4E-224	5,705712915	0,753	0,06	3,6676E-220	Pericytes	Sncg
101	8,3E-106	2,759845491	0,923	0,484	2,2377E-101	MSC_chondro	Mt1
102	4,1E-104	1,602038701	0,989	0,383	1,105E-99	MSC_chondro	Hp
103	2,1E-99	2,339912347	0,864	0,341	5,55135E-95	MSC_chondro	Mt2
104	4,97E-96	1,859691056	0,975	0,414	1,34259E-91	MSC_chondro	Cxcl14
105	2,01E-95	1,913768267	0,925	0,597	5,44474E-91	MSC_chondro	Zfp36
106	2,77E-94	1,515460758	0,998	0,624	7,48005E-90	MSC_chondro	Gpx3
107	1,84E-91	1,652352083	0,955	0,655	4,96966E-87	MSC_chondro	Fosb
108	2,22E-91	1,649460666	0,948	0,612	6,00015E-87	MSC_chondro	Egr1
109	2,13E-87	1,704394131	0,948	0,716	5,75263E-83	MSC_chondro	Zfp36l1
110	6,54E-86	2,656977014	0,658	0,182	1,76732E-81	MSC_chondro	Grem1
