# Supplementary material for: Heterogeneity of IL-15-expressing mesenchymal stromal cells controls natural killer cell development and immune cell homeostasis
Source: Nat Commun. 2025 Jul 1;16:5949. doi: 10.1038/s41467-025-61231-0 (PMC12218584; doi:10.1038/s41467-025-61231-0)
Supplement: Supplementary file 2 — Reporting Summary [file 41467_2025_61231_MOESM2_ESM.pdf]

nature portfolio

Corresponding author(s): Dietmar Herndler-Brandstetter
Last updated by author(s): Mar 12, 2025

# Reporting Summary

Nature Portfolio wishes to improve the reproducibility of the work that we publish. This form provides structure for consistency and transparency in reporting. For further information on Nature Portfolio policies, see our Editorial Policies and the Editorial Policy Checklist.

## Statistics

For all statistical analyses, confirm that the following items are present in the figure legend, table legend, main text, or Methods section.

| n/a | Confirmed | |
|---|---|---|
| ☐ | ☒ | The exact sample size (*n*) for each experimental group/condition, given as a discrete number and unit of measurement |
| ☐ | ☒ | A statement on whether measurements were taken from distinct samples or whether the same sample was measured repeatedly |
| ☐ | ☒ | The statistical test(s) used AND whether they are one- or two-sided<br>*Only common tests should be described solely by name; describe more complex techniques in the Methods section.* |
| ☒ | ☐ | A description of all covariates tested |
| ☐ | ☒ | A description of any assumptions or corrections, such as tests of normality and adjustment for multiple comparisons |
| ☐ | ☒ | A full description of the statistical parameters including central tendency (e.g. means) or other basic estimates (e.g. regression coefficient) AND variation (e.g. standard deviation) or associated estimates of uncertainty (e.g. confidence intervals) |
| ☐ | ☒ | For null hypothesis testing, the test statistic (e.g. *F*, *t*, *r*) with confidence intervals, effect sizes, degrees of freedom and *P* value noted<br>*Give P values as exact values whenever suitable.* |
| ☒ | ☐ | For Bayesian analysis, information on the choice of priors and Markov chain Monte Carlo settings |
| ☒ | ☐ | For hierarchical and complex designs, identification of the appropriate level for tests and full reporting of outcomes |
| ☒ | ☐ | Estimates of effect sizes (e.g. Cohen's *d*, Pearson's *r*), indicating how they were calculated |

*Our web collection on statistics for biologists contains articles on many of the points above.*

## Software and code

Policy information about availability of computer code

| | |
|---|---|
| Data collection | Flow cytometry data was acquired using FACS Diva Software (BD Biosciences).<br>Agarose gels were captured using a Bio-Rad ChemiDoc Imaging System and Image Lab Touch Software (Biorad).<br>Primers were designed using Primer3Plus Software.<br>scRNA-seq data were processed and aligned using Cell Ranger (7.1.0, 10x Genomics).<br>Animal husbandry and genotypes were documented using PyRAT Software (Scionics Computer Innovation).<br>qPCR data were processed using CFX Maestro (BioRad). |
| Data analysis | Flow cytometry data was analyzed using FlowJo V10 (BD Biosciences). Figures were plotted using Graphpad Prism and the R package ggplot2.<br>scRNAseq data were processed using Cell Ranger 7.1.0 (10x Genomics) and analyzed using R (4.4.1) and the packages Seurat (5.0.1), HGNChelper, harmony, ggplot2, scCustomize and paletteer.<br>qPCR data were pre-processed using CFX Maestro (BioRad) and analyzed using Microsoft Excel. |

For manuscripts utilizing custom algorithms or software that are central to the research but not yet described in published literature, software must be made available to editors and reviewers. We strongly encourage code deposition in a community repository (e.g. GitHub). See the Nature Portfolio guidelines for submitting code & software for further information.

## Data

Policy information about availability of data

All manuscripts must include a data availability statement. This statement should provide the following information, where applicable:

- Accession codes, unique identifiers, or web links for publicly available datasets
- A description of any restrictions on data availability
- For clinical datasets or third party data, please ensure that the statement adheres to our policy

Single cell RNA sequencing data generated in the present study have been deposited in the Gene Expression Omnibus (GEO) under accession no. GSE273212 (https://www.ncbi.nlm.nih.gov/geo/query/acc.cgi?acc=GSE273212). Accessible with the reviewer token: wjoxigsmthuxvmn

## Research involving human participants, their data, or biological material

Policy information about studies with human participants or human data. See also policy information about sex, gender (identity/presentation), and sexual orientation and race, ethnicity and racism.

| | |
|---|---|
| Reporting on sex and gender | *Use the terms sex (biological attribute) and gender (shaped by social and cultural circumstances) carefully in order to avoid confusing both terms. Indicate if findings apply to only one sex or gender; describe whether sex and gender were considered in study design; whether sex and/or gender was determined based on self-reporting or assigned and methods used. Provide in the source data disaggregated sex and gender data, where this information has been collected, and if consent has been obtained for sharing of individual-level data; provide overall numbers in this Reporting Summary. Please state if this information has not been collected. Report sex- and gender-based analyses where performed, justify reasons for lack of sex- and gender-based analysis.* |
| Reporting on race, ethnicity, or other socially relevant groupings | *Please specify the socially constructed or socially relevant categorization variable(s) used in your manuscript and explain why they were used. Please note that such variables should not be used as proxies for other socially constructed/relevant variables (for example, race or ethnicity should not be used as a proxy for socioeconomic status). Provide clear definitions of the relevant terms used, how they were provided (by the participants/respondents, the researchers, or third parties), and the method(s) used to classify people into the different categories (e.g. self-report, census or administrative data, social media data, etc.) Please provide details about how you controlled for confounding variables in your analyses.* |
| Population characteristics | *Describe the covariate-relevant population characteristics of the human research participants (e.g. age, genotypic information, past and current diagnosis and treatment categories). If you filled out the behavioural & social sciences study design questions and have nothing to add here, write "See above."* |
| Recruitment | *Describe how participants were recruited. Outline any potential self-selection bias or other biases that may be present and how these are likely to impact results.* |
| Ethics oversight | *Identify the organization(s) that approved the study protocol.* |

Note that full information on the approval of the study protocol must also be provided in the manuscript.

# Field-specific reporting

Please select the one below that is the best fit for your research. If you are not sure, read the appropriate sections before making your selection.

☒ Life sciences ☐ Behavioural & social sciences ☐ Ecological, evolutionary & environmental sciences

For a reference copy of the document with all sections, see nature.com/documents/nr-reporting-summary-flat.pdf

# Life sciences study design

All studies must disclose on these points even when the disclosure is negative.

| | |
|---|---|
| Sample size | No statistical method was used to predetermine sample size. Sample sizes were chosen based on standard experimental group sizes to achieve acceptable power taking into account the increased variability of animal models (i.e. 8-10 mice per group for in vivo experiments). |
| Data exclusions | No data were excluded. |
| Replication | Data from each genotype were from at least three independent experiments and at least three different litters. For scRNAseq, 3 individual sequencing runs were performed, for each of which multiple biological replicates were pooled. |
| Randomization | Group allocation was not randomized, PCR genotyping determined group allocation. Mice were processed in a random order and multiple investigators were involved in data generation and analysis. |
| Blinding | Blinding was not possible as the treatments were administered by the same party responsible for data collection. |

# Reporting for specific materials, systems and methods

We require information from authors about some types of materials, experimental systems and methods used in many studies. Here, indicate whether each material, system or method listed is relevant to your study. If you are not sure if a list item applies to your research, read the appropriate section before selecting a response.

## Materials & experimental systems

| n/a | Involved in the study | |
|---|---|---|
| ☐ | ☒ | Antibodies |
| ☒ | ☐ | Eukaryotic cell lines |
| ☒ | ☐ | Palaeontology and archaeology |
| ☐ | ☒ | Animals and other organisms |
| ☒ | ☐ | Clinical data |
| ☒ | ☐ | Dual use research of concern |
| ☒ | ☐ | Plants |

## Methods

| n/a | Involved in the study | |
|---|---|---|
| ☒ | ☐ | ChIP-seq |
| ☐ | ☒ | Flow cytometry |
| ☒ | ☐ | MRI-based neuroimaging |

## Antibodies

Antibodies used

antibody (clone) company fluorophore(s) cat. no
a4b7 (DATK32) Biolegend APC 120608
Bcl2 (BCL/10C4) Biolegend AF647 633510
CD3e (145-2C11), Biolegend BV785 100355
CD3e (500A2) Biolegend APC-Fire750 152308
CD4 (RM4-5) Biolegend AF700 100536
CD8a (53-6.7) BD Biosciences BB700 566410
CD8a (53-6.7) Biolegend PerCP, BV650 100732, 100742
Cd11b(M1/70) Biolegend FITC, PerCP 101230, 101206
Cd1d BV421 (1B1) 123527
Cd11c (N418) Biolegend APC-Fire750 117352
CD19 (1D3/CD19) Biolegend APC-Cy7 152412
CD24 (30-F1) Biolegend APC 138505
CD25 (3C7) Biolegend PE-Cy7 101916
CD27 (LG.3A10) Biolegend FITC 124207
CD31 (MEC13.3), Biolegend PE-Cy7 102523
CD44 (IM7) Biolegend FITC 103006
CD45 (30-F11) Biolegend AF700, APC-Fire750 103128, 103154
CD49a (HM1a) Biolegend PE-Cy7 142608
CD49b (DX5) Biolegend APC 108910
CD51 (RMV-7) Biolegend PE 104106
CD54 (YN1/1.7.4) Biolegend AF647 116120
CD62L (MEL-14) Biolegend PE-Cy7 104417
CD69 (H1.2F3) Biolegend PE 104507
CD71 (R17217) Biolegend APC-Fire750 113828
CD73 (TY/23) Biolegend PE 117204
CD106 (429) Biolegend AF647 105712
CD117 (2B8) Biolegend BV785 105841
CD117 (ACK2) Biolegend BV605 135122
CD122 (TM-β1) Biolegend PE-Cy7 123219
CD127 (A7R34) Biolegend PE 135009
CD135 (A2F10) Biolegend BV421 135314
CD144 (BV13) Biolegend BV421 138013
CD146 (ME-9F1) Biolegend PE 134704
CD200 (OX-90) BD Biosciences BV421 565547
CXCR3 (CXCR3-173) Biolegend BV421 126522
F4/80 (BM8) Biolegend PE 123110
Gr1 (Rb6-8c5) Biolegend APC-Cy7 108423
Ifng (XMG1.2) Biolegend APC 505810
Ki67 (11F6) Biolegend BV711 151227
Klrg1 (2F1/KLRG1) Biolegend APC 138412
LepR (polyclonal) R&D Systems Biotin BAF497
Ly6C (HK1.4) Biolegend PE-Cy7 128017
Ly6G (1A8) Biolegend FITC 127605
NK1.1 (PK136) Biolegend BV421 108732
NK1.1 (PK136) Biolegend purified 108702
NKG2D (CX5) Biolegend APC 115711
Sca-1 (D7) Biolegend FITC 108105
TCRβ (H57-597) Biolegend APC-Fire750 109246
Ter119 (Ter119) Biolegend APC-Cy7, FITC, BV421 116223, 116206, 116233

Tnfa (MP6-XT22) Biolegend FITC 506303
Mouse Fc block TruStain FcX PLUS (anti-mouse CD16/32) Biolegend / 156604

Validation

All antibodies were purchased from commercial sources. Biolegend and BD antibodies are tested by ELISA capture. Polyclonal anti-Leptin R antibody was tested by R&D in direct ELISAs and Western blots.

# Animals and other research organisms

Policy information about studies involving animals; ARRIVE guidelines recommended for reporting animal research, and Sex and Gender in Research

Laboratory animals

Il15GFP homozygous reporter knockin mice were generated by D.H.-B. in the lab of Richard A. Favell (Yale University, USA; MTA #20104). Il15flox/flox mice were generated by Nan-Shih Liao (Academia Sinica, Taiwan; MTA #13T-1050130-16M) and purchased from The Jackson Laboratory (Stock No. 034188). Cdh5-CreERT2 mice were kindly provided by Ralf H. Adams (Max Planck Institute for Molecular Biomedicine, Germany; MTA #016258). Prx1-Cre (Stock No. 005584), Lepr-Cre (Stock No. 008320), Osx1-GFP-Cre (Stock No. 006361), Ai14-Tomato (Stock No. 007914) and CXCL12-DsRed knockin/knockout mice (Stock No. 022458) were purchased from The Jackson Laboratory. Conditional knockout lines were generated by interbreeding Il15flox/flox with the respective Cre lines. Age- and sex-matched Cre- and Cre+ mice (littermates wherever possible, 8-16 weeks old if not explicitly stated otherwise) have been used in all experiments.

Wild animals

None used

Reporting on sex

Male and female mice were used equally.

Field-collected samples

None used

Ethics oversight

All experiments involving animals were approved by the animal ethics committee of the Medical University of Vienna and the Austrian Federal Ministry of Education, Science and Research (GZ 66.009/0407-V/3b/2018 and GZ 66.009/0408-V/3b/2018).

Note that full information on the approval of the study protocol must also be provided in the manuscript.

# Plants

Seed stocks

*Report on the source of all seed stocks or other plant material used. If applicable, state the seed stock centre and catalogue number. If plant specimens were collected from the field, describe the collection location, date and sampling procedures.*

Novel plant genotypes

*Describe the methods by which all novel plant genotypes were produced. This includes those generated by transgenic approaches, gene editing, chemical/radiation-based mutagenesis and hybridization. For transgenic lines, describe the transformation method, the number of independent lines analyzed and the generation upon which experiments were performed. For gene-edited lines, describe the editor used, the endogenous sequence targeted for editing, the targeting guide RNA sequence (if applicable) and how the editor was applied.*

Authentication

*Describe any authentication procedures for each seed stock used or novel genotype generated. Describe any experiments used to assess the effect of a mutation and, where applicable, how potential secondary effects (e.g. second site T-DNA insertions, mosiacism, off-target gene editing) were examined.*

# Flow Cytometry

## Plots

Confirm that:

☐ The axis labels state the marker and fluorochrome used (e.g. CD4-FITC).

☒ The axis scales are clearly visible. Include numbers along axes only for bottom left plot of group (a 'group' is an analysis of identical markers).

☒ All plots are contour plots with outliers or pseudocolor plots.

☒ A numerical value for number of cells or percentage (with statistics) is provided.

## Methodology

Sample preparation

BM cells were isolated from femur and tibia of 8 to 16 week old mice (if not explicitly indicated otherwise). For immune cell panels, BM was thoroughly flushed out of the bones with ice-cold FACS buffer (1x PBS, 2% FBS), centrifuged for 5 min at 400 x g and then incubated in 2mL ACK lysis buffer (150 mM NH4Cl, 10 mM KHCO3, 0.1 mM Na2EDTA, pH 7.4) for 5 min at RT to lyse erythrocytes. After a second centrifugation step, samples were resuspended in an appropriate volume of FACS buffer and Fc receptors were blocked with TruStain FcX Plus (Biolegend) before adding the specific surface antibodies for 30-60 min on ice. Samples were finally washed two times with 2 mL FACS buffer.
BM stromal cells were isolated using an enzymatic digestion protocol and handled in tubes pre-coated in FBS overnight at 4°C to reduce adherence to the plastic surfaces. Briefly, femurs and tibias were dissected from euthanized mice, and soft tissue was carefully removed. BM was flushed with staining buffer (PBS supplemented with 10% FBS and 2 mM EDTA) and the remaining bones were crushed using a mortar and pestle. The pelleted BM and bone fractions were then digested for 30-40 minutes at 37°C by using collagenase IV at 1 mg/mL (Sigma-Aldrich), DNase I at 1 mg/mL (Sigma-Aldrich) and Dispase at 0.5

mg/mL (Roche) in Dulbecco's Modified Eagle Medium (DMEM). The digestion mix was filtered through a 70 µm cell strainer to remove debris and bone fragments and neutralized with staining buffer. The filtered cell suspension was centrifuged at 300 × g for 8 minutes and resuspended in appropriate volume of staining buffer, Fc-blocked, stained, and washed.
To obtain splenocyte single cell suspensions, spleens were gently mashed using the flat end of a plastic 10 mL syringe and filtered through a 70 µm mesh cell strainer with 3 mL FACS buffer.
Peripheral blood was obtained by retro-orbital bleeding of isofluorane-anaesthesized mice, ACK-lysed twice for 5 min at RT and subsequently resuspended in FACS buffer.
In some instances, stromal cells were magnetically pre-enriched using the EasySep Mouse PE Positive Selection Kit (StemCell Technologies), e.g. for qPCR of CD73-PE+ Il15GFP+ and Il15GFP- sorted cells.

Instrument

BD LSR Fortessa X20 flow cytometer (BD Biosciences)

Software

BD FACSDiva software and FlowJo V10 (BD Biosciences)

Cell population abundance

Cell concentrations (BM of two femurs and tibias or whole spleen) were determined by counting trypan blue negative cells on a hemocytometer (Neubauer).

Gating strategy

All cells were gated on target cell size and granularity (FSC-A and SSC-A), single cells (FSC-H vs FSC-A / SSC-H vs SSC-A), live cells (Aqua Zombie or Ghost Dye negative). Following this quality gate, the gating strategies were employed according to the indications in the supplementary figures. In summary:
MSCs: Ter119-Lin- CD45- CD31- LepR+ VCAM-1+
Prx1-Tomato+ stromal cells: Ter119-CD71- CD31- CD45- Prx1-Tomato+
Hypertrophic chondrocytes: CD45- CD71-/TER-119-/CD31-/CD19- CD24+ CD200+
Proliferating chondrocytes: CD45- CD71-/TER-119-/CD31-/CD19- CD24+ CD200-
Sinusoidal endothelial cells: CD71-/TER-119- CD45- CD144+ CD31+ Sca1+ ICAM-1+
Arteriolar endothelial cells: CD71-/TER-119- CD45- CD144+ CD31+ Sca1hi ICAM-1-
LSK: CD3-/CD19-/Gr-1-/NK1.1-/TER-119- Sca-1+ CD117+
CLP: CD3-/CD19-/Gr-1-/NK1.1-/TER-119- CD117+ CD127+ Sca-1+ CD135+
rNKP: CD19-/Gr-1-/TER-119-/CD11c- CD3-NK1.1-CD135-CD27+CD122+CD127+
stage A iNK: CD19-/Gr-1-/TER-119-/CD11c- CD3-NK1.1-CD135-CD27+CD122+CD127-).
iILC1: CD45+Lin-NK1.1+CD3-CD127+CD49b-CD49a+
NKT: CD45+Lin-NK1.1+CD3+
mNK: CD45+Lin-NK1.1+CD3-CD49b+CD127-CD49a-
CD8+ TCM: CD45hiCD3+CD8+CD4-CD44+CD69-CD62L+
CD8+ TRM: CD45hiCD3+CD8+CD4-CD44+CD69+
CD8+ DCs: CD45+CD11c+CD8+
CD8- DCs: CD45+CD11c+CD8-
naïve macrophages: CD45+CD11b+F4/80+Ly6C-
inflammatory macrophages: CD45+CD11b+F4/80+Ly6C+

☒ Tick this box to confirm that a figure exemplifying the gating strategy is provided in the Supplementary Information.
